# Interdisciplinary Comprehensive Arm Rehabilitation Evaluation (ICARE): a randomized controlled trial protocol

**DOI:** 10.1186/1471-2377-13-5

**Published:** 2013-01-11

**Authors:** Carolee J Winstein, Steven L Wolf, Alexander W Dromerick, Christianne J Lane, Monica A Nelsen, Rebecca Lewthwaite, Sarah Blanton, Charro Scott, Aimee Reiss, Steven Yong Cen, Rahsaan Holley, Stanley P Azen

**Affiliations:** 1 Division of Biokinesiology and Physical Therapy, Herman Ostrow School of Dentistry University of Southern California, Los Angeles, California, USA; 2 Department of Neurology, Keck School of Medicine, University of Southern California, Los Angeles, California, USA; 3 Department of Rehabilitation Medicine, Emory University School of Medicine Center for Rehabilitation Medicine, Atlanta, GA, USA; 4 Department of Cell Biology, Emory University School of Medicine Center for Rehabilitation Medicine, Atlanta, GA, USA; 5 National Rehabilitation Hospital, Washington, DC, USA; 6 Georgetown University, Washington, DC, USA; 7 Washington DC VA Medical Center, Washington, DC, USA; 8 Statistical Consulting Research Center, Keck School of Medicine, University of Southern California, Los Angeles, CA, USA; 9 Department of Preventive Medicine, Keck School of Medicine, University of Southern California, Los Angeles, CA, USA; 10 Long Beach Memorial Medical Center, Long Beach, CA, USA

**Keywords:** Stroke, Brain infarction, Hemiparesis, Neurorehabilitation, Task-specific training, Motor recovery, Occupational therapy, Physical therapy

## Abstract

**Background:**

Residual disability after stroke is substantial; 65% of patients at 6 months are unable to incorporate the impaired upper extremity into daily activities. Task-oriented training programs are rapidly being adopted into clinical practice. In the absence of any consensus on the essential elements or dose of task-specific training, an urgent need exists for a well-designed trial to determine the effectiveness of a specific multidimensional task-based program governed by a comprehensive set of evidence-based principles. The Interdisciplinary Comprehensive Arm Rehabilitation Evaluation (ICARE) Stroke Initiative is a parallel group, three-arm, single blind, superiority randomized controlled trial of a theoretically-defensible, upper extremity rehabilitation program provided in the outpatient setting.

The primary objective of ICARE is to determine if there is a greater improvement in arm and hand recovery one year after randomization in participants receiving a structured training program termed Accelerated Skill Acquisition Program (ASAP), compared to participants receiving usual and customary therapy of an equivalent dose (DEUCC). Two secondary objectives are to compare ASAP to a true (active monitoring only) usual and customary (UCC) therapy group and to compare DEUCC and UCC.

**Methods/design:**

Following baseline assessment, participants are randomized by site, stratified for stroke duration and motor severity. 360 adults will be randomized, 14 to 106 days following ischemic or hemorrhagic stroke onset, with mild to moderate upper extremity impairment, recruited at sites in Atlanta, Los Angeles and Washington, D.C. The Wolf Motor Function Test (WMFT) time score is the primary outcome at 1 year post-randomization. The Stroke Impact Scale (SIS) hand domain is a secondary outcome measure.

The design includes concealed allocation during recruitment, screening and baseline, blinded outcome assessment and intention to treat analyses. Our primary hypothesis is that the improvement in log-transformed WMFT time will be greater for the ASAP than the DEUCC group. This pre-planned hypothesis will be tested at a significance level of 0.05.

**Discussion:**

ICARE will test whether ASAP is superior to the same number of hours of usual therapy. Pre-specified secondary analyses will test whether 30 hours of usual therapy is superior to current usual and customary therapy not controlled for dose.

**Trial registration:**

http://www.ClinicalTrials.gov Identifier: NCT00871715

## Background

Of the 795,000 individuals who will experience a new or recurrent stroke in the U.S. each year, a majority will have considerable residual disability [1-6]. Sixty-five percent of patients at 6 months are unable to incorporate the paretic hand effectively into daily activities [2,7]. In turn, this degree of disability contributes to a reduced quality of life after stroke [3,7-9]. The extent of disability has been under-represented by measures that capture only basic activities of daily living, such as self-care, and do not extend to activities and participation at higher levels of functioning that are most affected by a residual upper extremity disability [1,10-14]. Against this background, we designed and initiated the Interdisciplinary Comprehensive Arm Rehabilitation Evaluation (ICARE) Stroke Initiative. Although the proportion of stroke survivors who are mildly to moderately impaired is not definitively known, conservative estimates range between 5% and 30%. These are individuals who return to the community but with significant disability [15]. The paucity of dose-equivalent designs in the stroke upper extremity clinical trial literature (including the EXCITE (Extremity Constraint-Induced Therapy Evaluation) trial) [16], highlights the necessity and importance of this phase III RCT evidence [17,18].

The past decade has witnessed an explosion of different therapeutic interventions intended to capitalize on the brain’s inherent plasticity to increase adaptation to injury and learning into old age. The upper extremity (UE) interventions with the strongest evidence, and potentially the most immediate and cost-effective appeal for the current healthcare environment, share a common emphasis on focused task-specific training applied with an intensity higher than usual care [19,20]. Given this knowledge and considering the continuing constraints placed upon the total number of treatment hours for upper extremity rehabilitation, determining whether a program that is based both upon best practice and evidence-based interventions is superior to current care becomes imperative. Moreover, the value of such a program needs to be realistic given prevailing practice patterns; any superiority cannot simply be related to the amount of therapy provided. Such an intervention would incorporate a number of dynamic and competing processes with the following critical goals: 1) enable a balanced interaction between processes associated with experience-dependent and injury-induced cortical reorganization to best guide functional recovery [21-23]; 2) attenuate the detrimental effects of maladaptive compensatory strategies (e.g., learned non-use), often currently promoted during inpatient rehabilitation [2], that may with time persist and become more difficult for the patient and clinician to reverse [24]; 3) foster an early, but not too early, aggressive approach during a more vulnerable period both physiologically and psychologically [21,25,26]; and 4) overcome the challenges of introducing a principle-based, distributed, upper extremity task-specific training program into an already dwindling acute inpatient length of stay where UE use is frequently minimal [2,17,27].

ICARE is a randomized controlled trial (RCT) designed to compare ASAP, an integrated set of three essential elements (skill, capacity, motivation) bundled together in a theoretically defensible and reproducible protocol, to an equivalent dose of usual and customary outpatient therapy. To date, few upper extremity rehabilitation clinical trials have included dose equivalency in the therapy application trial design. The dose-equivalent control comparison is a particularly appropriate alternative given that: 1) the EXCITE trial design and findings did not rule out the possibility that usual and customary care provided at the same dose and intensity as constraint-induced movement therapy (CIMT) would have been as efficacious, 2) findings from the VECTORS (Very Early Constraint-Induced Movement during stroke rehabilitation) trial showed that a higher intensity of CIMT applied acutely after stroke was less efficacious, while a lower intensity of CIMT yielded comparable results to a dose-equivalent usual therapy group, and 3) well-designed investigations of upper extremity rehabilitation applied in the outpatient setting that compare the effectiveness of task-specific training to that of an equivalent dose of conventional therapy are sorely lacking [28-30] with one recent exception [31]. Finally, the non-dose-equivalent, observation only group (UCC) will help determine whether ASAP or DEUCC or both are superior to current clinical practice.

## Methods/design

We will test our hypotheses by randomizing 360 participants into a three center, single blind randomized controlled trial to investigate the effectiveness of a focused, intense, evidence-based, upper extremity rehabilitation program (ASAP) administered during the early post-acute stroke outpatient interval. Motor and quality of life measures of participants post-stroke randomized to ASAP treatment will be compared to those of participants administered an equivalent dose of usual and customary outpatient therapy (dose-equivalent usual and customary care, DEUCC), and an observation only usual and customary (UCC) occupational therapy control group. The primary study time point is 1 year after randomization. Our ultimate goal is to provide evidence to optimize post-stroke rehabilitation practice for those with mild to moderate upper limb impairments and reduce disability in the broadest sense.

All procedures conducted during this trial with human participants are carried out in compliance with federal and institutional ethical standards and in compliance with the Helsinki Declaration. All research procedures are approved by an Institutional Review Board at each of the participating sites: University of Southern California Health Sciences Campus Institutional Review Board (protocol #HS-07-00148), Emory University Institutional Review Board (protocol #IRB00001180), MedStar National Rehabilitation Hospital/MedStar Health Research Institute (protocol #2007-161), Rancho Los Amigos National Rehabilitation Center Institutional Review Board (protocol #037), Casa Colina Centers For Rehabilitation Institutional Review Board (protocol #HS-07-00148), Long Beach Memorial Care Health System Institutional Review Board (protocol #428-07), Huntington Memorial Hospital Institutional Review Board (protocol #HMH 2007–049), and Cedars-Sinai Institutional Review Board (protocol #Pro00012032).

### Type of design

**Figure 1:**
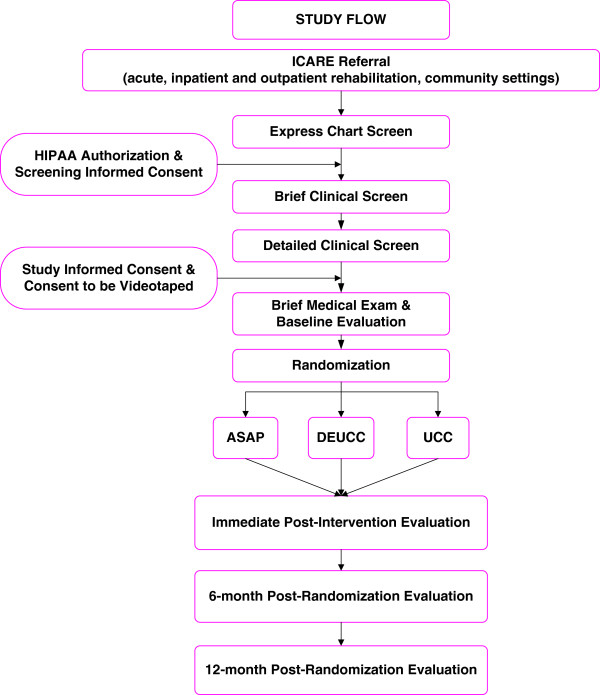
**Study flow from referral to follow-up. **The primary endpoint is 1 year after randomization.

ICARE is a parallel group, three arm, single blind, phase III, superiority, randomized, controlled trial of a principle-based upper extremity training program provided in the outpatient setting after the acute hospitalization phase. Once discharged from an inpatient or acute setting and following baseline assessment, participants are randomized, no earlier than 14 days and no later than 106 days after stroke onset, to one of three intervention groups: ASAP, DEUCC or UCC. The primary outcome, collected at 1 year post-randomization, is the log WMFT time score. The secondary outcomes are the SIS hand domain subscale and the full SIS scores. To prevent unintended crossover, details of the ASAP protocol are currently embargoed; therapists who provide ASAP sign a confidentiality and nondisclosure agreement and do not provide UCC or DEUCC treatments. Outcome assessors are blinded to treatment group. The study flow is illustrated in Figure 1.

### Study enrollment

Each center (University of Southern California, Emory University, and National Rehabilitation Hospital/Georgetown University) is expected to randomize 120 individuals. To confirm eligibility, pre-screening, screening, a brief medical examination and baseline evaluation are administered at one of the seven ICARE clinical sites. Eligible individuals who have provided informed consent are randomized following a baseline evaluation. Enrollment is defined by having signed a Study Informed Consent. The purpose and timing of events from pre-screening to randomization are summarized in Table 1.

**Table 1:** Pre-screening to randomization flow with purpose and time interval

**STUDY PHASE**	**EVENT**	**PURPOSE**	**TIME INTERVAL**
**PRE-SCREENING**	Express Chart Screen (ECS)	Chart review for excludable non-modifiable criteria is performed under IRB-approved HIPAA waiver.	Before 106^th^ day post-stroke
**SCREENING**	HIPAA Authorization & Screening Informed Consent (IFC)	Prospective Participants (PP’s) who pass Pre-Screen are introduced to the study. A signed HIPAA Authorization & Screening IFC are required to proceed.	
	Brief Clinical Screen (BCS)	An initial brief screen is completed to ensure sufficient motor and cognitive recovery and pre-morbid function for eligibility. If recovery is not sufficient, PP’s may be re-tested up to 106 days post-stroke.	
	Detailed Clinical Screen (DSC)	If BCS is passed, a detailed clinical screen for eligibility is administered. PP’s Primary Care Physician is notified if DCS is passed. Signed HIPAA authorization is included with the notification letter.	
**STUDY INFORMED CONSENT**	Study IFC & Consent To Be Videotaped	PP’s who pass DSC are informed about the study in greater detail. A signed Study IFC and Consent to be Videotaped define enrollment and are required to proceed.	
**BASELINE**	Brief Medical Exam At Baseline (BME)	Medical exam releases PP to participate in trial. Rules out interim neurologic event and serves as re-check for severe depressive symptoms. Opportunity for SPI to establish blood pressure, heart rate, weight-bearing and other medical parameters for safe participation.	
	Baseline Evaluation	Confirms UE motor eligibility. Establishes baseline measures for testing hypotheses.	Within 72 hours post-BME^1^ and between 14–106 days post-stroke.
**RANDOMIZATION**	Group Assignment	Treatment group assignment	≤ 48 hours post-Baseline and between 14–106 days post-stroke

^1^Ideally the Baseline Evaluation will occur within 72 hours post-BME (Brief Medical Exam). If more than 72 hours has transpired, participant must be re-cleared of an interceding neurologic event. PP = Prospective participant; ECS = Express Chart Screen; BCS = Brief Clinical Screen; IFC = Informed Consent; DCS = Detailed Clinical Screen; SPI = Site Physician Investigator.

### Accessing and collecting personal health information

In ICARE, both HIPAA Waiver and Authorization are employed in a two-tiered process: a) pre-screening; and b) screening, to assure privacy protection while minimizing prospective participant burden. Prospective participants are first identified through a review of admissions to the inpatient rehabilitation and acute medical units affiliated with ICARE clinical sites or by direct referral. Stroke diagnosis is initially confirmed by ICD-9 code, census review with the treating stroke teams, or the direct referral source. For those individuals with a confirmed stroke diagnosis, a chart review for non-modifiable exclusionary criteria (e.g. age, stroke characteristics, co-morbidities) is performed. Individuals passing the express chart screen are approached for the initial in person consented screening phase.

### Informed consent

ICARE uses a two-step consenting process; participants are asked to consent to a screen, and if qualified, a second consent is obtained for full study participation. Once a potential participant has been determined to qualify for screening, s/he is asked to sign the HIPAA Authorization and Screening Informed Consent to participate in a brief and detailed screen to determine study eligibility.

### Screening process

The pre-screening and screening processes are completed so that randomization occurs no later than 106 days post-stroke onset according to protocol. The pre-screen, or Express Chart Screen (ECS), is a chart review of non-modifiable eligibility criteria (Tables 1 &2). The screening process occurs in two phases: 1) Brief Clinical Screen (BCS) and 2) Detailed Clinical Screen (DCS). Candidates who pass the BCS are followed by the Clinical Site Coordinator (CSC), and administered the DCS at an individually determined time, adjusted to maximize the candidate’s potential for eligibility. Candidates who pass the DCS are offered the study informed consent and videotape consent. Enrollment commences with a signed Study Informed Consent. Upon receipt of these consents, the participant is scheduled for a Brief Medical Exam (BME) and Baseline Evaluation to be performed just prior to randomization. Eligibility criteria, pre-identified as likely to change during the screening phase (i.e., motor function, medical stability, and desire to participate), are confirmed at the BME and Baseline Evaluation.

**Table 2:** Eligibility criteria

	**INCLUSION**
1.	Ischemic or hemorrhagic stroke (subdural and epidural effusions permitted) within the previous 106 days
2.	Hemiparesis (weakness) in arm or hand
3.	Some active finger extension movement by close of enrollment window
4.	Age 21+
5.	Able to communicate in English
6.	Willing to attend outpatient therapy and all study evaluations
	**EXCLUSION (truncated list)**
	*Neurologic symptoms or conditions*
1.	Traumatic or non-vascular brain injury, subarachnoid hemorrhage, AV malformation, acute subdural or epidural hematoma
2.	Neurologic condition that may affect motor response (e.g. Parkinson’s, ALS, MS)
3.	Presence of ataxia per NIHSS [32] and evidence of cerebellar or brainstem lesion
4.	Absent upper extremity sensation per NIHSS
5.	Neglect asymmetry > 3 per Mesulam Unstructured [33]
6.	A second stroke within the last 72 hours cannot be ruled out before the brief medical exam (BME)
	*Physical attributes affecting movement or function*
1.	Total UE Fugl-Meyer score <19 or >58, or = 0 for finger mass extension/grasp release hand score
2.	UE pain that substantially interferes with ADL’s
3.	Maximum assistance required for mobility
4.	Passive ROM limitation of the hemiparetic upper extremity that prevents functional use of limb/hand, including any of the following:
	1. Shoulder: flexion <90°, abduction <90°, external rotation <45°
	2. Elbow/Forearm: extension <−20°, supination or pronation < 45° from neutral
	3. Wrist/Finger: flexion or extension <0°, MCP or IP extension <30°
	*Pre-morbid status*
1.	Head trauma requiring > 48 hours of hospitalization within past 12 mos.
2.	Psychiatric illness requiring hospitalization within past 24 mos.
3.	Arm or hand injury limiting use prior to stroke
4.	Amputation of all fingers or thumb of affected hand
5.	Pre-morbid motor impairment of the contralateral upper extremity of neurologic origin
6.	Barthel Index [10] < 95
	*Medication, Drug and/or Alcohol*
1.	Active or recent drug treatment for dementia
2.	Treated with Botox in affected arm within last 3 months
3.	Toxicology screen positive for illegal substances or reported use within the past 3 years
4.	Reported alcohol use per CAGE or treatment for withdrawal since index stroke
	*Cognition and Participation*
1.	Enrollment in a conflicting study
2.	Expected inability to participate in study due to illness, social, or geographic reasons
3.	Unable to follow a 2-step command per NIHSS
4.	< 2 on the Mini-Cog [34] with an abnormal Clock Draw Test (CDT) [35] or score = 0
5.	PHQ-9 total score between 10 and 19 without management plan or score >19
6.	Judged medically unstable and/or unable to participate by primary physician or SPI
	*Other*
1.	Received > 6 hours of Outpatient Occupational Therapy (OT) since stroke (Home Health and OT Evaluation do not count toward 6 hour maximum)
2.	Clinician’s best judgment (multiple factors in combination): The SPI and CSC concur that the PP is NOT a candidate for randomization

If a participant fails a specific screening criterion, he/she is deemed ineligible for the study. Initial failure of certain criteria that are likely to change during this dynamic period of recovery, such as motor function, sensation, or depressive symptoms, does not necessarily lead to ineligibility. The Clinical Site Leadership team (CSC and Site Physician Investigator (SPI)) has the option of withholding a decision of disqualification in lieu of re-screening during the eligible post-stroke period. If all eligibility criteria are passed at the Baseline Evaluation, the participant is randomized. Essential details of the ECS, BCS, DCS, BME and Baseline Evaluation prior to randomization are detailed in Table 1 along with the time interval within which these events are performed.

### Inclusion and exclusion criteria

#### Stroke diagnosis

Participants are adults with diagnosis of ischemic stroke or intraparenchymal hemorrhagic stroke. CT or MRI scans are used to confirm stroke diagnosis. If a scan is not available, the diagnosis is confirmed by clinical criteria. Specific Inclusion and exclusion criteria are provided in Table 2.

### Assessments

#### Outcome measures

There are four evaluation points in ICARE: baseline, completion of intervention, and 6 months and 1 year after randomization (Figure 1). The primary time point at 1 year is used to test the primary and secondary hypotheses using the analysis plan described herein.

The log-transformed WMFT time score at the 1 year endpoint is the primary outcome measure for the trial and the basis of *a priori* sample size and sensitivity estimates. The SIS hand domain and full SIS constitute the secondary outcome measures. The WMFT and SIS are described in detail below. A full list of assessments is included in Table 3, which describes the timing for data acquisition by each instrument including the WMFT and SIS. The full battery of assessments is designed to provide information about muscle strength, cognition, digit sensation-perception, functional ability, depression, self-efficacy, life satisfaction, reintegration, and subjective quality of life. These are listed in the Additional file 1 and arranged roughly into categories using the International Classification of Functioning and Disability Framework [36] (ICF).

**Table 3:** Baseline and follow-up assessments for each participant at each time point

**Outcome Assessment**	**Baseline**^**1**^	**Post-Intervention**^**2**^	**6-mo Post Randomization**^**3**^	**12-mo Post Randomization**^**4**^
Patient Health Questionnaire, (PHQ-9) [37,38]	X^5^	X	X	X
NIH Stroke Scale (NIHSS) [32,39-41]	X^5^	X	X	X
WMFT, 15 timed items	X	X	X	X
WMFT, 2 strength items	X	X	X	X
WMFT Functional Ability Scale, 15 items (WMFT- FAS)[42,43]	X	X	X	X
Stroke Impact Scale (SIS)	X	X	X	X
Cognitive Battery – 5 items	X			X
· Short Blessed Memory Orientation & Concentration Test[44]	X			X
· D-KEFS Verbal Fluency Test[45]	X			X
· Hopkins Verbal Learning Test, Revised[46]	X			X
· Digits Span Backward	X			X
· Color Trails 1 & 2 [47,48]	X			X
Upper Extremity Fugl-Meyer, Motor (UEFM) [49,50]	X	X	X	X
Satisfaction with Life Scale (SWLS)[51-53]	X	X	X	X
Arm Muscle Torque Test [54]	X	X	X	X
AsTex Sensory Index [55]	X	X	X	X
Motor Activity Log, 28 item (MAL-28)[56]		X	X	X
EQ5D [57]	X	X	X	X
Confidence in Arm & Hand Measure (CAHM)	X	X	X	X
Reintegration to Normal Living Index (RNLI) [58,59]	X	X	X	X
Single-Item Quality of Life Measure (SQOL) [60,61]	X	X	X	X
Physiologic Measures	X	X	X	X
Monthly Telephone Follow-up^5^		X	X	X
Post-Intervention Exit Interview^5^		X		
Final Study Exit Interview^5^				X

Unless otherwise specified, assessments are administered and scored by standardized BE’s.

^1^Randomization occurs after baseline and between 14–106 days post stroke; thus participant and all study personnel are blinded to treatment group assignment at the baseline evaluation.

^2^Post-Intervention Evaluation occurs between 16–20 weeks post-randomization.

^3^6-month Post-Randomization Evaluation occurs between 24–28 weeks post-randomization.

^4^12-month Post-Randomization Evaluation occurs between 50–64 weeks post-randomization.

^5^Items at given time points are measured by local site team personnel and not by BE’s.

Trained licensed occupational or physical therapists blinded to group assignment (Blinded evaluators, BE) and certified for administration, perform all evaluations. The primary outcome assessment is filmed using a digital video camera according to detailed procedures outlined in the Manual of Procedures (MOP). Identical computer, digital video camera, editing, compression and conversion software are standard equipment that were issued to each ICARE site during the start-up phase. A FTPS (File Transfer Protocol Secure) server is maintained to enable digital video review and standardization and data file transfer across sites and to the trial’s administrative team.

#### Wolf motor function test (WMFT)

The WMFT is a laboratory-based functional assessment of the arm and hand [62]. The instrument includes 15 measures of speed (timed tasks), 2 measures of strength (arm, grip), and 15 measures of movement quality using the Functional Ability Scale (FAS) of the upper extremity [63].The FAS movement quality score is determined from video review by a panel of raters blinded to treatment group and assessment time point. Items include a hierarchically arranged set of functional movements of the shoulder, elbow and hand that progress from proximal movements to distal hand tasks including grasp control and manipulation. The WMFT has been shown to be both reliable and valid in the stroke population [64,65]. It has been used previously, in numerous studies including the EXCITE and VECTORS trials.

#### Stroke impact scale (SIS)

The Stroke Impact Scale (SIS) 3.0 is a well-established 59-item interviewer-administered assessment of health-related quality of life for individuals after stroke [66,67]. The SIS captures 8 domains of function organized into subscales as well as an overall measure of perceived recovery from stroke. Both the full SIS 3.0 (ICF Participation level) and the hand function subscale (ICF Activity level) are used in ICARE as secondary outcome measures. The hand function subscale requires participants to indicate the difficulty experienced over the past 2 weeks with 5 activities involving the paretic hand, including: carrying heavy objects, turning a doorknob, opening a can or jar, tying a shoelace, and picking up a small coin.

### Other assessments

#### Monthly follow-up interviews

Within 30 days of randomization, the recruiting site team initiates monthly telephone interviews with each participant to ascertain information about health status, healthcare utilization, medications, other therapies, and adverse events.

#### Post-intervention interview

A multiple question survey interview is administered by a non-treating, unblinded member of the recruiting team upon completion of the intervention phase. At the post-intervention evaluation, each participant is asked a set of questions to assess the extent to which critical components of the investigational intervention (e.g. impairment mitigation, session intensity, participant chosen tasks, therapist-participant collaboration) were incorporated into each assigned therapy group [68].

#### Exit interview

At the final 1 year evaluation, participants complete another set of questions regarding activity since the end of the intervention phase. Participants are also asked to report the perceived value of the intervention and study participation.

### Usual care evaluation and treatment records

Receipt of usual and customary care, defined as outpatient occupational therapy (OT), is monitored and recorded from stroke onset to study endpoint. No more than six hours of outpatient occupational therapy treatment, excluding an OT initial evaluation, is permitted prior to randomization. Usual care in treatment hours is recorded at the time of randomization. Once randomized and through the end of the intervention observation window (16 weeks post-randomization), usual care is recorded at an individual session level for all treatment groups. Data include: 1) Date, 2) Length of treatment session in minutes, 3) Content, using CPT® codes, 4) Payer, 5) Location (at an ICARE site (on-site) or other therapy locations (off-site)), and 6) Change in (OT) prescriptions. Additional data collected include: initial prescription, all missed appointments and reason for absence, discharge and discharge reason. From these data, usual care dose, therapy content, attendance behaviors and reimbursement mechanisms can be used for secondary analyses. For therapy occurring off-site, all post-stroke outpatient OT records are procured with a signed HIPAA authorization. For therapy on-site, the Study Informed Consent and HIPAA authorization signed at screening permit internal access to these records. From the outpatient therapy records, the usual care data are gleaned during the intervention observation window. When a participant is randomized, he or she is provided with a monthly calendar up to the study endpoint and instructions to record all therapies attended, noting the date, time spent in therapy and type of therapy. These calendars are reviewed monthly during the telephone interview with the participant and provide the data collected regarding ancillary therapy utilization as well as outpatient OT received outside of the intervention observation window.

### Medication information

Data about medication prescription and usage are collected during pre-screening, screening, prior to baseline and at monthly intervals post-randomization. Prior to baseline, receipt of tPA (if known) is recorded and all prescribed medications are recorded by name. A participant-specific medication list is developed at the time of the Baseline assessment. Monthly, this list is revisited with the participant and updated accordingly. New medications are added at this time and data regarding discontinued medications are recorded. These data are intended to provide supplemental information about the medical and pharmacologic management of the ICARE sample.

### Standardization of assessments

All clinical research blinded evaluators (BE’s) must complete an initial certification (standardization) process for the WMFT and SIS, and maintain re-certification at least every 6 months, thereafter. A 3-day training workshop was conducted during the start-up phase, serving to introduce the BE’s to the standardized test protocols. Updated training materials include: printed manuals of test administration and scoring instructions; online video demonstration; peer review; practice with volunteers who have had a stroke; monthly training meetings; and an online discussion board. Certification is granted for performance of at least 90% mastery. The certifying therapist views a digital recording of test administration and scoring by the therapist seeking certification along with all assessment-specific study documentation; detailed verbal and written feedback are provided. All study personnel administering the National Institutes of Health Stroke Scale (NIHSS) [32,39,40] must obtain certification of standardized competency.

#### WMFT FAS rater panel

A panel of 3 experienced therapists provide FAS ratings for the WMFT. Inter-rater reliability is refined via teleconferences. The Data Management and Analysis Center (DMAC) convert and compress the site submitted edited digital WMFT test administration. Media stream access is provided to the members of the rater panel through the secure ICARE database website, thereby enabling remote viewing and scoring capability. Intra-rater reliability and inter-rater reliability were initially assessed using a sample of 8 videotaped WMFT test administrations procured from a database of previous studies. Raters rated each video at that time and after 1 month. A web/teleconference followed to review and discuss discrepant individual item ratings. This resulted in further refinement of the scoring guidelines. Two additional test-retest iterations were completed, each time with a set of 21 filmed ICARE WMFT test administrations. These were analyzed using weighted Kappa statistics, Spearman correlation coefficients and Bland-Altman plotting to determine points of disagreement. Currently, each of the 3 panel members rate each video independently, to yield 3 independent ratings per video. Specific instances of item score disagreement are identified by the DMAC for review and discussion by the panel before adjudication by majority vote. The WMFT FAS Panel meets via tele-conference monthly to view and rate a new video as a group.

### Randomization method

A stratified block randomization schema is used for each site to balance randomization assignment by motor severity and time from stroke onset. Participants are stratified using an ordered set of variables defined by: 1) baseline motor impairment using the Fugl-Meyer Upper Extremity motor score [49,69,70] (more impaired ≤ 35, less impaired ≥ 36, eligible range 19–58); and 2) days from stroke onset (early ≤ 59 days and late 60+ days, eligible range 14–106). No *a priori* assumptions are made about there being balance across stratification factors. Randomization assignment is obtained through the secure web-based data entry system, which confirms that all prerequisites have been completed before informing the CSC of the assignment. Each CSC maintains a randomization log at the site, and the DMAC maintains the master list for the trial. The balance of the group assignment is monitored weekly by the DMAC, and reported to the DSMB quarterly, with treatment group coded to maintain blinding.

### Interventions

#### Accelerated skill acquisition program

**Figure 2:**
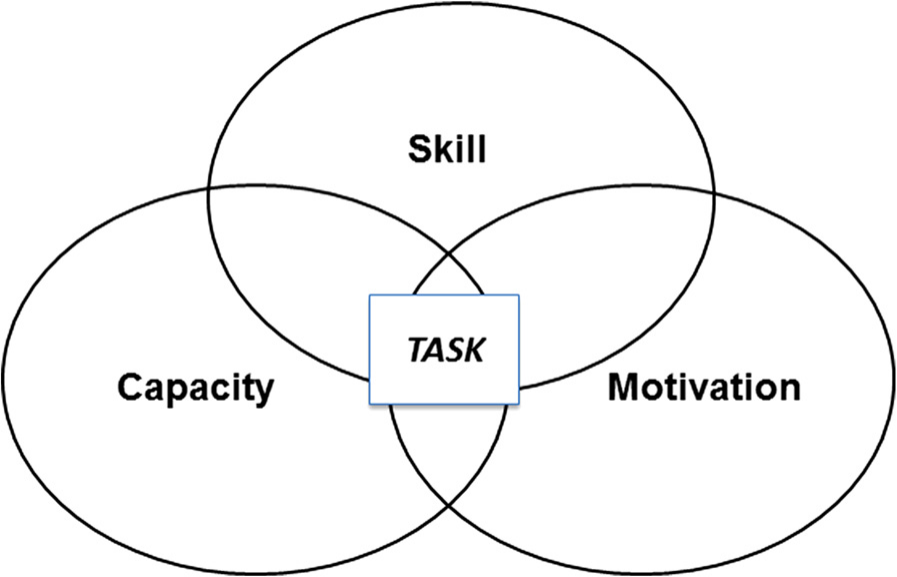
**Conceptual model of the Accelerated Skill Acquisition Program (ASAP).** The conceptual model reflects ASAP’s intersecting emphases on skill acquisition, capacity building (impairment mitigation), and motivational enhancement. Challenging movement tasks are used as vehicles to address neurorehabilitation and recovery.

The Accelerated Skill Acquisition Program (ASAP) is a fully defined, principle-based protocol that integrates three fundamental elements including: **skill** acquisition through task-specific practice, impairment mitigation to increase **capacity**, and **motivational** enhancements to build self-confidence (Figure 2). ASAP is grounded in the evidence-based expectation that effective rehabilitation of the paretic upper extremity is achievable and based upon the provision of challenging, intensive, and meaningful task practice for motor skill acquisition, mitigation of associated linchpin impairments and dysfunctions of movement, and the confidence to integrate use of emerging skills into daily life activities [2,19,20,71,72].

The ASAP structured protocol includes an initial evaluation and orientation session (Session A) and 30 1-hour visits of an individualized practice program focused on function of the arm and hand most affected by the stroke. This integrated and evidence-based intervention for upper extremity recovery arose from diverse but converging, complementary, and interdisciplinary literatures of basic and applied science as well as recent translational and stroke clinical trial research of upper extremity recovery. Unique aspects include the structured framework by which intensity and progression of practice is managed and which fosters participant skills and confidence through therapist-patient collaboration. Participants randomized to ASAP are given a customized package of therapy that includes challenging, intensive, and meaningful practice of activities related to participant chosen real-world tasks (e.g., carrying groceries, handwriting) that engage the arm most affected by the stroke. Participants are offered a mitt to wear on the less affected hand during the time outside of therapy to promote use of the weaker arm and hand; however, the participant is not required to use the mitt if so chosen. Outside of therapy specific assignments (i.e., Action Plans) are given to encourage self-managed, confident, safe, and effective arm use at home and in the community.

Table 4 summarizes the eight evidence-based, non-exclusive (over-lapping) operating principles that are currently used to guide ASAP intervention sessions: 1) ensure challenging and meaningful practice [20,73-76], 2) address important mutable impairments through task-breakdown [77-80], 3) enhance motor capacity through overload and specificity [81,82], 4) preserve natural goal-directedness in movement organization [83,84], 5) try to avoid artificial task breakdown when engaging in task-specific practice [85], 6) assure active patient involvement and opportunities for self-direction [86,87], 7) balance immediate and future needs for efficient motor skill and capacity enhancement with the development of confidence and self-management skills [88,89] and 8) drive task-specific self-confidence (self-efficacy) high through performance accomplishments [90,91]. Together, these principles are designed to emphasize and drive the development of skilled movement performance.

**Table 4:** ASAP’s eight overlapping operating principles

	**Principle**
1	Ensure challenging and meaningful practice
2	Address important (interfering) changeable impairments
3	Enhance motor capacity through overload and specificity
4	Preserve natural goal-directedness in movement organization
5	Avoid artificial task breakdowns when possible
6	Assure active patient involvement and opportunities for self-direction
7	Balance immediate and future needs
8	Drive task-specific self-confidence high through performance accomplishments

#### ASAP protocol parameters

The program begins with an orientation and evaluation session to accomplish the following 10 goals: 1) prepare the collaborative ‘real-world’ task list to be used during training; it includes 6 tasks the patient most wants to perform with at least two bimanual activities, two strength-dependent activities including the most affected arm, and two activities requiring dexterity of the most affected hand, 2) designate a priority or benchmark task from the collaborative task list, 3) determine fundamental impairments and the priority-task challenge threshold or movement breakdown point(s), 4) prepare a collaborative schedule for the first day of training, 5) orient the participant to the mitt and its function including safety precautions, 6) identify appropriate conditions for mitt wearing and not wearing, 7) orient the participant to a recurring brief self-efficacy question, 8) orient the participant to out-of-lab action plans, 9) orient the participant to their collaborative role with the trainer and 10) obtain the participant’s agreement to be a collaborative partner. The orientation and evaluation Session A may last up to two full hours.

Training sessions are to be 1 hour per day, 3 times per week for a total of 30 hours, with rest breaks allowed to maximize engaged time-on-task. Following a debriefing of the previously assigned Action Plan and the acquisition of several physiologic measures (i.e., BP, heart rate, pain, muscle soreness), each regular training session begins with a collaborative ordering of the real-world tasks identified during Session A (orientation and evaluation). The real-world tasks may change as interests and goals evolve over the 30 sessions, however the priority task may not change for benchmarking purposes. Task and movement analysis are done for each real-world task to determine the key movement dysfunctions or impairments. The goal of intervention training is to focus attention and effort directly on the problematic area (i.e. dysfunction, impairment) to facilitate skill acquisition without simply providing a compensatory strategy as an easy and quick fix to the problem. Classic exercise-overload principles (e.g. intensity, periodicity) are used to drive progression and build motor capacity (e.g., muscular strength and endurance, coordination). Practice activities within the three categories of real-world functional tasks (i.e., strength, dexterity, bimanual) are selected based on patient task preferences. Training is collaborative and interactive with the participant providing problem identification as feasible (e.g., “What is the limiting factor when you perform that task?”) and solutions through self-assessment and trainer feedback/suggestions. Confidence building and empowerment are embedded in the training and education during each session. Task-specific (i.e., priority task) self-efficacy assessment is performed 4 times throughout the 30 sessions using a Brief Self-Efficacy Rating Scale. The participant is asked to provide a number between 0 and 10 in response to the question: “How confident are you that you can (fill in specific priority task activity)?” The confidence rating is followed by a question asking, “What can we do this week to increase your capabilities and raise your confidence?” As the participant gains insight and develops self-assessment and management skills, the response to the follow-up question is expected to become more insightful and rich in self-knowledge and ways to drive challenge and improvement. One strategy for building capacity is to perform inter-session ‘Action Plans’ or out of clinic activities. All 30 sessions have associated Action Plans including some with handouts that provide background information related to ASAP principles and evidence. Table 5 lists the knowledge-based handouts provided to participants and the planned distribution order across the 30 sessions. Individual participant-specific Action Plans are also generated and used throughout the 30 sessions. These Action Plans encourage specific practice in the home or community settings. Examples include finding a challenging task involving food preparation or eating or reading an education handout about motor recovery. At the beginning of each session, participants are asked to report on the effectiveness of their Action Plan activities on the next day of training. The therapist also rates the participant’s engagement in the Action Plan on a scale from 1 = no effort/did not attempt, 2 = some effort, and 3 = great effort/engagement, apparently practiced diligently.

**Table 5:** List of standard action plans with handouts

1.	Using the soft mitt on the better hand
2.	Preventing arm and hand injuries while training
3.	Family and friend support
4.	Helpful thoughts for performing challenging activities in public
5.	Getting the most learning for your efforts (Motor learning Part I)
6.	Turning science into skill (Motor learning Part II)

**Figure 3:**
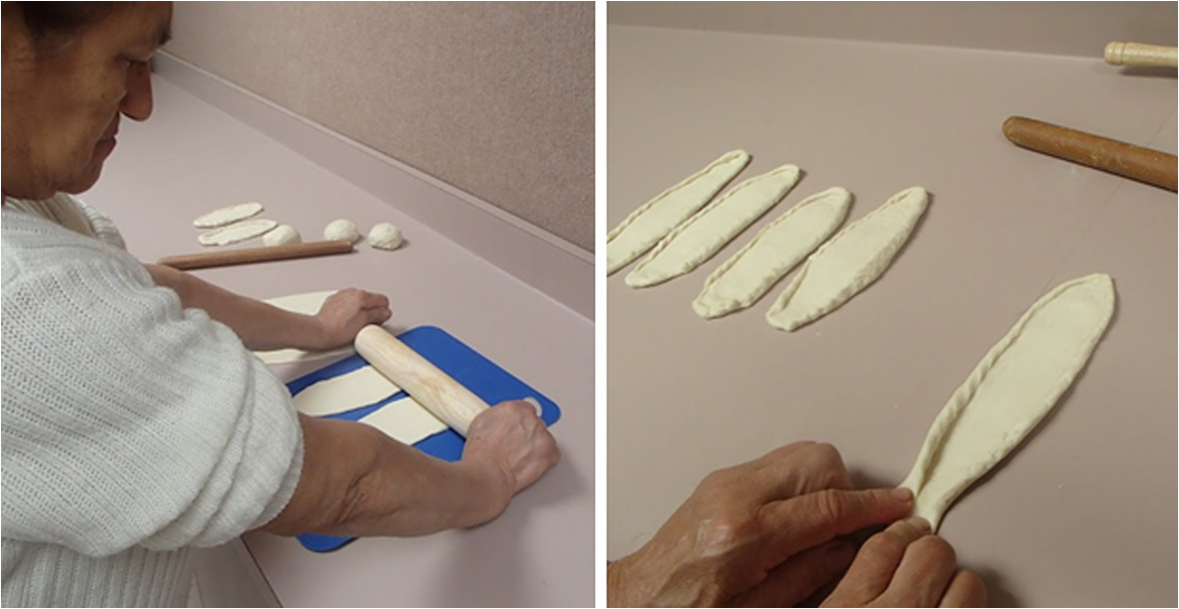
**Examples of two participant-selected tasks.** These photographs depict a participant engaged in two sensorimotor tasks, each addressing different motor impairments and movement skills. The study participant selected these two tasks during her ASAP sessions for practice.

Figure 3 illustrates the clinic environment and two examples of tasks selected by participants to practice during ASAP sessions. To prevent cross-contamination between study groups, further details of the investigational intervention as outlined in the ASAP MOP have been embargoed until study completion.

#### Standardization for the ASAP protocol

To ensure data quality and consistent administration of the intervention among all ASAP therapists, a staged standardization process (Phase 1 and 2, Phase 3 and Re-certification) was developed. At each stage, an expert reviewer (experienced members of the ICARE Investigative team) assesses the digital video footage accompanying documentation for therapist mastery of each ASAP principle implemented during a 1 hr session. Phase 1 and 2 standardizations must be attained working with a volunteer with stroke before a therapist may initiate therapy with a randomized participant in the ICARE trial. Once Phase 1 and 2 standardizations have been attained, the therapist may begin working with an ASAP-randomized participant to complete Phase 3 standardization. After Phase 3 standardization is acquired, the therapist must re-certify according to the schedule directed by the reviewer. If in the worst case scenario, a therapist fails to demonstrate ASAP treatment fidelity at Phase 3 certification or recertification thereafter, all post-randomization data accrued for a participant seen by that therapist will be designated unusable for outcome analysis.

The Phase 1 and 2 standardization processes include demonstration of mastery in the following three task areas: 1) administration of the Brief Self-Efficacy Question; 2) determination of the challenge threshold and fundamental movement problem for two arm and hand activities, and 3) determination of appropriate task practice intensity and activity/task progression. Additional mastered elements for Phase 1 and 2 include: generalized ASAP protocol awareness via a knowledge test and therapist self-assessment of at least 90% mastery. Phase 3 standardization is attained via demonstrated mastery of the ASAP protocol through review of the 1^st^ and 20^th^ treatment sessions administered to the new therapist’s first ASAP randomized participant. Recertification is maintained through demonstration of continued mastery, on a target schedule of approximately every 6 months. During the trial start-up period, a 3-day training workshop was held for all ASAP therapists to attain Phase 1 standardization. Intervention therapists in all stages of standardization and the protocol developers facilitate ongoing training via monthly ASAP therapist teleconferences with video and literature review, individual conferencing, an on-line discussion board, and site visits for digital video review and discussion of ASAP principles and clinical experiences.

#### Usual and customary care (UCC)

The UCC group receives Outpatient Occupational Therapy for the upper extremity as determined by each participant’s individual occupational therapist, based upon usual and customary practice standards. In accordance with those practices, the participant attends a standard Occupational Therapy evaluation session prior to initiating intervention sessions (treatment). The number of visits and frequency (dose) for the intervention is determined per the therapy prescription generated at the evaluation, site-specific usual and customary practices, and payer guidelines (e.g. private insurance, HMO, Medicare). The evaluation may have occurred prior to randomization. The dose may be modified per usual and customary practices as the intervention ensues. The treating therapist(s) documents each session per the treating facility’s protocol and documentation methods. The CSC of the clinic that randomized the participant is responsible for procuring the evaluation and treatment records and gleaning the necessary information for ICARE using standardized forms. Vital signs, as well as any symptoms of fatigue or stress are monitored during each session in accordance with usual and customary standard practice.

#### Dose-equivalent usual and customary care (DEUCC)

The DEUCC group initially receives Outpatient Occupational Therapy for the upper extremity as determined by each participant’s individual therapist, based upon usual and customary practice standards in a manner identical to the previously described UCC group with respect to content. Differentiating this group from UCC is its dose-equivalency with the ASAP group; each receives 30 hours of distributed treatment. Prior to initiating treatment (intervention) and in accordance with usual and customary practices, the participant receives a standard Occupational Therapy evaluation, where a recommended number of visits and frequency (dose) are determined, in accordance with site-specific usual care practices. This evaluation may have occurred prior to randomization. Immediately following the initial evaluation, the therapist and patient are notified of group assignment and 30 sessions are scheduled. According to protocol, if more than 30 treatment sessions are scheduled, the CSC will indicate that the treatment window is closed and a post-test will be scheduled. Provision of further treatment is documented via the monthly telephone interviews. Documentation and data are collected in a manner identical to that for the UCC group.

#### Dose of therapy (ASAP and DEUCC)

Thirty hours of training was chosen for the ASAP protocol because this dosage fell within the range of previous rehabilitation intervention trials that were shown to be effective in the post-acute outpatient setting. A training schedule of 30 hours of treatment, ideally distributed into 1 hour sessions, 3 days per week over 10 weeks is the anticipated experimental frequency for both the DEUCC and the ASAP groups. Both interventions vary in content and structure only. Pilot data from a multi-site outpatient survey conducted prior to initiating ICARE suggested that 30 hours of training would be higher than that commonly prescribed in the outpatient post-acute setting, and the 1 hour session length was designed to afford practicality and allow patients to participate in other concurrent therapy services (e.g., physical therapy and speech therapy). Those randomized to DEUCC or ASAP are expected to receive at least 90% (≥ 27 hours) of the 30 scheduled hours between 10 to 16 weeks post-randomization to be considered adherent to the dosing protocol. Therapy may extend up to 16 weeks to compensate for delays in initiating treatment post-randomization, missed sessions due to illness or other unavoidable absences. Precise tracking of therapy dose is maintained on all participants including: frequency and duration of individual treatment sessions, duration of total treatment sessions, and number of missed and canceled appointments. Additionally, for the UCC and DEUCC groups, initial OT prescription, prescription change, treatment venue and change in treatment venue are documented.

### Statistical analysis

#### Sample size and participant accrual

Sample size estimates were computed for the primary *a priori* aim of detecting a clinically relevant difference in log-transformed WMFT time score between ASAP and DEUCC at 1 year follow up with α = 0.05. With the proposed total sample of 360, we would have sufficient power to detect a moderate difference in treatment effect of Δ = 0.40 for an attrition rate of 17%, and Δ = 0.42 for an attrition rate of 25% using a two-group 2-sided t-test with a type I error of 0.05 and 80% power. The EXCITE trial showed treatment effect sizes of 0.50 and 0.63 for the high and low functioning groups, respectively. For the small sample phase II VECTORS study, a similar analysis showed a treatment effect size of 0.20. Given the outpatient timing of ICARE, a less dynamic period of change than VECTORS, we expect to have sufficient power to detect the effect size in log WMFT time score. We also should have sufficient power to detect a difference in the proportion of participants who change ≥ 25 points on the normalized SIS at 1 year using a χ^2^ statistic. The minimal success rate difference that can be detected with 80% power ranges from 13.5-20% (with 17% attrition) to 14.0-21% (with 25% attrition); the variation in the estimates reflects potential variation in improvement in the DEUCC group. To achieve this recruitment goal (N = 360) in 40 months, each of the three centers aims to randomize 3 participants per month.

### Specific aims

#### Primary Aim and hypotheses

##### Specific Aim 1

To compare the efficacy of a fully-defined, evidence-based and theoretically defensible therapy program (ASAP) and an equivalent dose of usual and customary Occupational Therapy (DEUCC) initiated within the earliest post-acute outpatient interval (14–106 days post stroke) for significant gains in the primary outcome of paretic upper extremity function 1 year after randomization.

##### Hypothesis 1

At 1 year post randomization, the time score from the WMFT will be significantly smaller (faster) after ASAP than usual and customary occupational therapy care (DEUCC), controlled for dose.

##### Secondary hypothesis for SA 1

At 1 year post randomization, the proportion of patients with successful outcomes measured by the Stroke Impact Scale (SIS) hand domain and full SIS will be greater after ASAP than DEUCC, controlled for dose.

#### Secondary Aim and hypotheses

##### Specific Aim 2A

To compare the efficacy of a fully-defined, evidence-based and theoretically defensible therapy program (ASAP) to that of an observation only usual and customary (UCC) occupational therapy program initiated within the earliest post acute outpatient interval (14–106 days post stroke) for significant gains in the primary outcome of paretic upper extremity function 1 year after randomization.

##### Hypothesis for SA 2

At 1 year post randomization, the time score from the WMFT will be significantly smaller (faster) after ASAP than UCC, uncontrolled for dose.

##### Secondary hypothesis for SA 2A

At 1 year post randomization, the proportion of patients with successful outcomes measured by the SIS hand and full SIS will be greater after ASAP than UCC, uncontrolled for dose.

##### Specific Aim 2B

To compare the efficacy of one dose-equivalent usual and customary outpatient occupational therapy program (DEUCC) to an observation only, usual and customary outpatient occupational therapy (UCC) program initiated within the earliest post-acute outpatient interval (14–106 days post stroke) for significant gains in the primary outcome of paretic upper extremity function 1 year after randomization.

##### Hypothesis for SA 2B

At 1 year post randomization, the time score from the WMFT will be significantly smaller (faster) after DEUCC than UCC, uncontrolled for dose.

##### Secondary hypothesis for SA 2B

At 1 year post randomization, the proportion of patients with successful outcomes measured by the SIS hand domain and full SIS will be greater after DEUCC than for UCC, uncontrolled for dose.

### Primary statistical analyses

#### Baseline

Descriptive statistics of demographics, baseline characteristics, and distributions will be performed for the total sample and for each group. Group comparisons will be made to examine whether any of these variables need to be accounted for in further analysis due to an imbalance across groups. Continuous variables will be compared using ANOVA for normally distributed variables to test for mean differences, and Wilcoxon rank sum test will be used for non-normally distributed variables to test for median differences. χ^2^ or Fisher’s exact test will be used for categorical variables to test for frequency differences. Participant characteristics that differ at baseline will be included as covariates in the analysis of the primary and secondary outcomes.

#### Analysis plan

This trial addresses two different questions separated by primary aim and secondary aim: 1) Is ASAP superior to DEUCC (primary aim)?, and 2) Is ASAP or DEUCC superior to UCC, (secondary aim) For all analyses, assumptions required for the data distribution (e.g., normal distribution) will be assessed. Any transformations of data or alternative methods necessary to analyze the data will be determined by examining the structure of the data. All analyses will be performed in accord with the intent-to-treat (ITT) principle (e.g., group status is determined by randomization at baseline). A p-value < 0.05 will be used to indicate statistical significance for all analyses.

The primary aim analyses will compare the ASAP and DEUCC group change in log WMFT time score and success rate from the SIS hand domain and the full SIS. For the primary hypothesis, change in WMFT time score at 1 year, an independent sample t-test will be used to compare the mean change in log-transformed WMFT time score, and analysis of covariance (ANCOVA) will be used to adjust for *a priori* covariates (site, initial motor impairment, stroke duration to randomization, baseline log time score) and any baseline variables that were imbalanced between ASAP and DEUCC. For missing data from loss to follow-up or missed evaluations at 12-months, multiple imputation using all available evaluation data will be utilized. Adjusted least-square means and the associated 95% confidence interval will be presented as well as p-value, and effect sizes will be computed to aid in interpretation of results.

For the secondary hypothesis, the success rate of the SIS hand domain will be calculated as the percent of subjects in the ASAP and DEUCC groups that achieved at least a 25-point increase in normalized SIS hand function at 1 year post randomization compared to baseline. Logistic regression will be used to compare these rates between groups, while adjusting for covariates. Since there are no prior data or biological evidence suggesting that ASAP will have a differential effect on one subgroup compared to DEUCC in the targeted population, we are not planning any *a priori* interactions between treatment and any of the covariates in the main analysis. However, we will perform exploratory data analysis to examine these possible interactions and generate hypotheses for future studies.

The analytic approach to the secondary aims will follow that described for the primary aims above, with the exception of comparing differences between groups receiving 30 hours of treatment (ASAP and DEUCC) to UCC. For other secondary outcomes, baseline to 1-yr change will be compared between ASAP and DEUCC groups and ASAP and UCC groups using analysis of covariance or logistic regression models (see Additional file 1). In addition, a composite physical domain, which includes strength, hand function, ADL/IADL, and mobility, will be created and compared in a similar way. Bonferroni adjustments will be made of the critical p-value for these secondary analyses, as there are two comparisons: ASAP to UCC and DEUCC to UCC; a p-value < 0.025 will be used to indicate significance.

#### Interim analysis plan

A single interim data analysis of Specific Aim 1 (ASAP vs DEUCC) for only the primary outcome, WMFT time, will be performed when approximately 33% of the participants have completed the 1-year evaluation. The O’Brien-Fleming group sequential method [92] will be used with the significance level defined as 0.005 at the interim analysis to maintain the overall type I error of 0.05. The advantage of the O’Brien Fleming method is: 1) it is conservative for significance at the interim analysis point, and 2) it maintains the conventional p-value in the final analysis. Group assignment will remain blinded with codes X and Y representing either ASAP or DEUCC groups to avoid revealing treatment assignment. Only the primary outcome for the primary aim (log WMFT mean time) will be analyzed as described above. If the test of success rate in WMFT time at the interim analysis point reaches a p-value less than these critical p-values, the DSMB will decide whether analysis of secondary outcomes is necessary to provide additional support of the results. If deemed necessary by the DSMB, conditional power will be computed to make a reassessment of the power at the end of the trial.

#### Missing data

The DMAC conducts weekly checks for all expected measurements in the database. Missing data must be identified by the cause of missing data, (e.g. participant not able to complete the measurement or evaluator error, forgot to record the data). Detailed reasons for missing data are recorded in a comment box under each measurement. This missing data form/point control procedure will enable future analysis for missing data patterns (e.g., systematic or random), and thus, support decision making for final data analysis.

### Adverse event monitoring and reporting

Adverse events (AE) are monitored closely and supported by the ICARE database that sends auto-alerts to team members, the NINDS clinical trial liaison to the DSMB and the Medical Safety Monitor (MSM). AEs include new events not present prior to enrollment in the study or events that were present during the pre-enrollment period but have increased in severity. All adverse events are reported in terms of three factors: 1) Serious or Non-serious, 2) Expected or Unexpected, 3) Related or Unrelated. These discrete categories and each characteristic of the event are evaluated independently of the others. An AE is considered serious (SAE) if it involves death, a life-threatening event, inpatient hospitalization, or persistent and/or significant disability/incapacity that lasts more than 48 hours, limits activities of daily living, and (in the opinion of the investigators) represents significant hazard or potential serious harm to the participant or others. Non-serious events are minor events that do not seriously limit a participant’s activities of daily living or present a potential risk to the participant or others. Expected serious events include: recurrent stroke or TIA; myocardial infarction or acute coronary syndrome; new onset of cardiac arrhythmia; fracture; pulmonary embolism; inpatient hospitalization or ER visit greater than 23 hours; and death. Expected non-serious events include: fall with no fracture; dyspnea; open sore or cuts; muscle soreness or pain that persists for more than 48 hours; shoulder pain that limits study participation; excessive blood pressure responses that require treatment discontinuation for the day; dizziness/fainting; deep vein thrombosis, without pulmonary embolus, and depression requiring mental health intervention. A related adverse event reflects a realistic chance of a causal relationship between participation in the study and the adverse event as suggested by an event that follows within a reasonable time after research procedures (i.e., 24 hours), follows a pattern consistent with study procedures, improves when study procedures have stopped and/or reappears when the procedures in question are repeated. An adverse event that does not reflect a realistic chance of a causal relationship between participation in the study and the adverse event, as described above for a related adverse event, is an unrelated adverse event.

#### Adverse events reporting procedures

The adverse event reporting system was developed with NINDS guidance, and has a pre-planned, automatic work-flow that triggers instant electronic communication. A site team member completes the online “Adverse Event Initial Report Form” within 24 hours of knowledge of an event and in compliance with all local IRB reporting procedures. This initiates the report process. The initial report is instantly added to the ICARE database and triggers an immediate, automatic notification to the study PIs, project manager, originating site’s physician investigator and clinical site coordinator, and DMAC members. The site physician investigator is responsible to obtain all necessary facts to verify and adequately describe the event, determine seriousness, determine the appropriate course of action and report these items via the ICARE “Adverse Event Confirmation Form” to the database within 72 hours after the initial report submission. If the AE is confirmed as serious, an immediate, automatically triggered email notification with a narrative of the SAE is sent to the study PI’s, project manager, originating site’s physician investigator and clinical site coordinator, MSM and the DSMB liaison. The MSM will review the SAE narrative and adjudicate study-relatedness and expectedness within 5 days (120 hours) of the automated email notification of an SAE. If data are not entered within the first 96 hours, an automated courtesy reminder is sent to the MSM. A detailed AE report is generated weekly and available to all study personnel. An AE summary report is generated and reviewed quarterly by the Data Safety and Monitoring Board (DSMB) and the USC IRB. Each site reports to its respective IRB in accordance with its policies and procedures. All AE reports, and amendments to them, are recorded and monitored by the DMAC.

### Data management and quality

#### Data management

The DMAC consists of the director, who is the unblinded statistician and who is responsible for managing the DMAC and acting as liaison to the DSMB for data-related matters, including preparation of reports and analyses (interim and primary outcomes). The other DMAC members are staff from USC’s Statistical Consultation and Research Center and include a co-director in charge of database development and management, a programmer, data manager and two research assistants. Additionally, a blinded statistician serves on the executive committee and is able to answer statistical questions that arise without danger of breaking the blind of the study.

#### Quality control procedures

Microsoft® SQL Server 2000 Edition is utilized for the secure database. This database is configured to utilize transaction logging for auditing and recovery purposes. All data entry/retrieval travels over the Hypertext Transfer Protocol Secure (HTTPS) layer. Backups are conducted daily, weekly, monthly and quarterly. The backup files are stored in a backup server at a secure separate location. The WMFT video files are stored in a multimedia server and streamed to the data entry system with the corresponding accession number, which provides blinding to site and evaluation time point.

Users are issued individual usernames and passwords to gain access to the secure web-based data entry system. Since ICARE is a multisite study, each site has access only to its site- specific data records. Within each site, only designated personnel can enter and modify data. The blinded evaluators are prohibited from accessing any treatment related information in the database, including randomization assignment.

All clinical report forms (CRF) are barcoded to prevent ID error. Sites are required to scan and submit the original CRFs for all key outcome measurements to the DMAC for data entry quality checking. Quarterly, the DMAC performs data quality checking (QC) on 100% of primary outcome data (WMFT and SIS).

### Study organization and management

ICARE is conducted by an experienced multidisciplinary research team, and is managed through a series of committees. This trial uses a multiple-PI management plan with the following designations: PI#1 is Carolee Winstein, PhD, PT, FAPTA, PI#2 is Alexander Dromerick, MD, and PI#3 is Steven Wolf, PhD, PT, FAPTA. PI#1 chairs the executive committee (EC), the small study leadership group that guides ICARE implementation and operations. This committee is composed of the PIs, study project manager (PM), blinded statistician and DMAC consultant, and NINDS scientific program director. It meets weekly by conference call. The EC is responsible for the general design and conduct of the study, protection of human research subjects, review of study progress and data collection, changes in study procedures, allocation of resources, and communication with the DSMB and NINDS. All dissemination ideas are vetted through the Executive Committee. When necessary the EC invites the DMAC Director, to participate in the meeting especially for preparation and discussion of DSMB reports. PI#1, 2 and 3 lead implementation of the scientific agenda and specific aims and ensure that systems are in place to guarantee responsible management of the RCT.

PI#1 serves as the primary contact PI, assumes fiscal and administrative responsibility for the trial and plays a lead role in the training and standardization of the ASAP therapists, including development of multi-media training tools. The DMAC, in consultation with PI#1 and the PM, develop and provide all reports to the DMSB and the NIH in accord with a pre-established time line for reporting.

PI#2 is responsible for all medical and neurologic issues that arise during participant screening, therapy, and follow-up. As study medical director, he is responsible for identification of stroke diagnosis and characterization and inclusion/exclusion criteria related to medical and diagnostic features of study participants. This responsibility includes review of brain images and response to diagnostic queries, review of adverse events and communication with the DSMB liaison when appropriate.

PI#2 and 3 are responsible for managing their subcontract and providing leadership at their respective Center including all clinical affiliates (PI#2 NRH; PI#3 Emory) to assure that each center investigative and clinical team implement the trial in accord with all policies and procedures detailed in the MOP. Additional leadership at each geographic center is provided by a designated center coordinator. Each site is led by a Clinical Site Coordinator (CSC) and Site Physician Investigator (SPI). For each of the roles of CSC, SPI, intervention therapist and blinded evaluator, there are trained, standardized back-up personnel. An experienced clinician oversees training and certification for the WMFT, with consultation from the tool’s developer (PI#3) and the PM. All PIs, the PM and DMAC Director share in monitoring study progress, including: recruitment, adherence, retention, safety and missing data. The Executive Committee guides the management of issues identified study-wide; whereas PI#1, the PM and, when necessary, the DMAC Director, manage site-specific issues. Both groups collaborate with the site teams to implement solutions and monitor effectiveness. PI#1, the PM and DMAC Director visit each site annually to monitor data quality, process and compliance and consult regarding site-specific issues. PI#1 and the PM coordinate with the DMAC on website and informatics work, to issue weekly progress reports to the investigative and clinical teams, and to maintain and update the MOP and CRFs as needed.

The Data Safety and Monitoring Board (DSMB) is appointed by the NINDS to ensure highest quality and ethical study execution and consists of a stroke neurologist, a physical therapist, a rehabilitation neurologist, a biostatistician, and an NINDS DSMB liaison. The DSMB evaluates a full study report, prepared by the DMAC Director and Project Manager, biannually. This report provides a summary of study activities, issues and progress since the last report in narrative form followed by data tables and figures, which include grouped data sets, blinded for treatment allocation. As an appendix to the report, each site prepares a site-specific narrative including proactive strategies for any areas of concern. Both the main report and the site-specific reports address the primary issues of: participant accrual, execution of intervention adherence (ASAP & DEUCC only), retention, safety and data quality, including missing data, protocol adherence, and safety. An abbreviated report is evaluated by the DSMB during the interceding quarters. The DSMB monitors serious adverse events and reviews cumulative adverse event data quarterly to ensure study safety. These data are also grouped, but blinded for treatment allocation. The DMAC Director prepares unblinded reports and analyses if requested by the DSMB. The DSMB meets biannually with the Executive Committee, DMAC Director, and MSM, alternating between tele-conference and in person meetings per annum. Additional meetings may be scheduled at the discretion of the DSMB or NINDS.

An independent Medical Safety Monitor (MSM) is chosen by the NINDS staff and the Executive Committee. This rehabilitation physician is responsible for timely review and adjudication of all serious adverse events and for reviewing summary reports of all adverse events on a regular basis. The MSM attends the annual DSMB meetings as well as the quarterly phone meetings.

The Clinical Site Coordinator (CSC) Committee consists of the Project Manager, and seven Clinical Site Coordinators; a DMAC member, PI#1 and other study personnel join as needed. This committee is responsible for collaborative training and problem-solving; recruitment, protocol implementation; data entry; resolution of site differences and assurance of trial consistency. This committee meets twice monthly.

The Clinical Research Committee is comprised of PI’s #1, 2 and 3, the PM, the three Clinical Center Coordinators (CCC), the DMAC Director and the psychosocial consultant. It meets monthly and is responsible for strategic planning, protocol development and implementation, CRF development and identification and development of secondary research questions.

The Physician Investigator Committee consists of the seven physician investigators across the clinical sites, the CCC, the PM, and PI#1. This group addresses relevant medical issues, assists in study recruitment, develops strategies to enhance recruitment, and facilitates acquisition of neuroimaging studies for secondary analyses. This committee will generate topics for secondary publications relevant to ICARE and the medical management of sub-acute stroke.

A Blinded Evaluator Committee consists of all blinded evaluators across the three centers, the CCC, PM and the WMFT reviewer. The BE committee focuses on standardization and execution of measurement, assurance of data completeness, participant safety, and prevention and documentation of unblinding.

The Publications Committee is comprised of the three PIs and the DMAC Director. It will establish a policy and oversee study publications to ensure that they are ethical and of high scientific quality. The committee will mediate when there is disagreement about an issue related to the form or scope of the publication and related issues including authorship. While generally not responsible for decisions regarding content, the committee may intervene in cases where a manuscript may include claims or interpretations that are not supported by the data. All publications emergent from the ICARE database will be vetted through the publications committee.

## Discussion

ICARE will contribute significantly to the practice of outpatient stroke rehabilitation, our knowledge of arm and hand recovery and to our understanding of how to conduct large-scale, multi-site clinical trials of complex rehabilitation interventions. There will be several unique contributions to the practice of arm and hand therapy after stroke. First, ICARE tests the value of a structured, principle-based and standardized arm and hand rehabilitation program in a sufficient number of participants of modest variety to provide a reasonable basis for confident inference to the clinical practice setting. Second, the primary endpoint, one year after randomization, assesses the durability of any benefit at a time sufficiently removed from the therapy to reflect behavioral learning (i.e., patient-centered control) rather than a short-term performance boost that may be therapist and/or therapy dependent.

The value of ASAP will be tested against an equivalent dose (30 hours) of usual and customary therapy delivered by an Occupational Therapist that provides a plausible control for the number of outpatient visits during a time of dwindling rehabilitation services. The trial will also help to determine the current state of outpatient therapy services for post-stroke arm and hand rehabilitation as there is a monitoring-only usual and customary care comparison group for which the prescribed dose is monitored. Finally, the trial will examine the impact of treatment on a sufficient number of ancillary assessments that cut across the ICF domains to make its outcomes easily comparable with those of other trials.

### Current study status

Enrolling participants. We expect to close enrollment by early 2013. An interim analysis was completed in November, 2011. The DSMB recommended continuation of recruitment.

## Abbreviations

RCT: Randomized Controlled Trial; ASAP: Accelerated Skill Acquisition Program; DEUCC: Dose-Equivalent Usual and Customary Care; UCC: Usual and Customary Care; DSMB: Data Safety and Monitoring Board; DMAC: Data Management and Analysis Center; SAE: Serious Adverse Event; CRF: Clinical Report Form; MSM: Medical Safety Monitor; CCC: Clinical Center Coordinator; CSC: Clinical Site Coordinator; BE: Blinded Evaluator; SIS: Stroke Impact Scale; WMFT: Wolf Motor Function Test; FAS: Functional Ability Scale; PM: Project Manager; ICF: International Classification of Functioning, Disability and Health; CIMT: Constraint Induced Movement Therapy; BME: Brief Medical Exam; BCS: Brief Clinical Screen; DCS: Detailed Clinical Screen; SPI: Site Physician Investigator; ECS: Express Chart Screen; AVM: Arteriovenous Malformation; UEFM: Upper Extremity Fugl Meyer; MOP: Manual of Procedure; BP: Blood Pressure; TPA: Tissue Plasminogen Activator; ITT: Intention-to-treat; CPT® codes and descriptions: contained in the CPT®/Medicare Relative Value Payment File are copyright 2009 American Medical Association. All rights reserved. CPT® is a registered trademark of the American Medical Association (AMA) http://www.ama-assn.org/ama/pub/physician-resources/solutions-managing-your-practice/coding-billing-insurance/cpt.shtml .

## Competing interests

The authors declared that they have no competing interests.

## Authors’ contributions

CJW in collaboration with SLW and AWD led the conceptualization, design, and implementation of this research protocol. CJW is one of two founding developers of the ASAP principles and protocol being tested in this trial. She chairs the executive committee and is the primary author for this manuscript. SLW is responsible for managing his subcontract and providing leadership to his Center including all clinical affiliates. He provides guidance to the work of the WMFT FAS review panel and BE committee with respect to the WMFT. He is a contributing author and provided critical review of the manuscript. AWD is the study Medical Director for ICARE. He is responsible for all medical and neurologic issues that arise during participant screening, therapy, and follow-up. He chairs the Physician Investigator Committee. He is responsible for managing his subcontract and providing leadership to his Center including all clinical affiliates. He is a contributing author and provided critical review of the manuscript. CJL directs the DMAC and is responsible for data management and primary analysis; she wrote statistical portions and data management portions of this manuscript. MAN is the project manager. She provides leadership in the design and implementation of this research protocol across all work groups and clinical sites and chairs the Clinical Research Committee. She wrote portions of this manuscript and contributed significantly to the editorial process. RL is an investigator and one of the two founding developers of the ASAP principles and protocol. She chairs the ASAP therapist committee. She wrote portions dealing with ASAP and several of the outcome measures and provided insightful and critical review of the entire manuscript. SB is a center coordinator (Emory) and clinical site coordinator. She participates in recruitment, screening, and treatment of participants, and contributed to protocol development/ revisions. She is a contributing author to this manuscript. CS is a clinical site coordinator (Long Beach), clinical center coordinator (California), and ASAP Intervention therapist. She chairs the CSC committee and is a contributing author of this manuscript. AR is a blinded evaluator and participates in the recruiting, screening and evaluating of study participants. She is a contributing author to this manuscript. SYC co-directs the DMAC and is responsible for database development and management. He wrote portions of the manuscript dealing with data management and adverse events. RH is the former center coordinator (NRH), clinical site coordinator at NRH and ASAP intervention therapist. He is a contributing author to this manuscript. SPA is the blinded statistician and DMAC consultant who serves on the Executive Committee. He was involved in proposal preparation and is a contributing author to this manuscript. All authors read and approved the final manuscript.

## Pre-publication history

The pre-publication history for this paper can be accessed here:

http://www.biomedcentral.com/1471-2377/13/5/prepub

## Supplementary Material

Additional file 1 **Outcome Assessments listed in Table **3**categorized using the International Classification of Functioning and Disability Framework.** Click here for file
