# Supplementary material for: Interdisciplinary Comprehensive Arm Rehabilitation Evaluation (ICARE): a randomized controlled trial protocol
Source: BMC Neurol. 2013 Jan 11;13:5. doi: 10.1186/1471-2377-13-5 (PMC3547701; doi:10.1186/1471-2377-13-5)
Supplement: Additional file 1 — Outcome Assessments listed in Table 3categorized using the International Classification of Functioning and Disability Framework. [file 1471-2377-13-5-S1.docx]

**Supplemental Material**

**Appendix:** Additional Outcomes listed in **Table** 3

**Body Function/Body Structure Level Measures:**

***NIH Stroke Scale***

The National Institutes of Health stroke scale (NIHSS) is one of the major stroke impairment scales used to quantify neurologic deficits after stroke [[1-4](#_ENREF_1)]. This 13 item test measures several aspects of brain function, including consciousness, vision, movement, sensation, speech, and language. The NIHSS has a high inter-rater reliability and is thus very appropriate for clinical treatment trials in which different persons may perform serial assessments. Study personnel responsible for administering the NIHSS require certification to administer this examination.

Portions of the NIHSS are administered during the screening phase while the entire scale is used as an outcome assessment. Items # 5, 7 & 8 are administered during the BCS. At Baseline, the entire examination is administered by the SPI or other licensed site personnel under the supervision of the Physician Investigator. The NIHSS at this time point serves as a baseline measure, a recheck of eligibility and an aid to the site physician in ruling out a new neurological event. It is reassessed at each follow-up evaluation.

***Arm Muscle Torque Test***

Upper extremity strength is measured in kilograms via maximum isometric torque testing in six isometric positions using the digital hand-held Lafayette manual muscle test dynamometer, model #001163 and standard testing positions as described by Andrews et al [[5](#_ENREF_5)]. Each of six muscle groups (flexors and extensors of the shoulder, elbow and wrist) are tested three times bilaterally during a 3 second isometric contraction with a 1-minute rest between trials. The greatest torque measured for each muscle group is used. Prior to testing each muscle group, the motion is demonstrated and the participant is passively taken through the arc of motion that would be generated if s/he were not isometrically stabilized. Verbal encouragement is provided throughout each trial. Each muscle group is tested in a gravity-eliminated position with the participant in a supine position. The shaft of the dynamometer is held perpendicular to the tested limb segment while the body is manually stabilized proximal to the tested limb segment and the participant is asked to push maximally against the curved plate and the piston of the hand-held device. Each dynamometer is to be calibrated annually and in accordance with the manufacturer’s recommendations. The arm muscle torque test is administered at each evaluation time point.

***Upper Extremity Fugl-Meyer (Motor)***

The UE Fugl-Meyer (UEFM) motor section includes tests of reflexes, active motion, and coordination [[6-8](#_ENREF_6)]. The upper extremity motor section is comprised of 33 tasks, with a maximum score of 66, and measures reflexes, volitional movement including flexor/extensor synergies, movement combining synergies, movement out of synergy, stability and movement of wrist and hand, and coordination/ speed. All movements are graded on a three point ordinal scale (0 = movement cannot be performed, 1 = movement is partially performed, 2 = movement is performed fully and equally to the opposite upper extremity). Movements are performed on the less affected upper extremity first, then the more affected upper extremity. Higher scores indicate better movement abilities. The UEFM is performed as part of the screening process to determine appropriate arm and hand movement eligibility and given at each evaluation period.

***Patient Health Questionnaire-9 (PHQ-9)***

The Patient Health Questionnaire- PHQ-2 and PHQ-9 are two versions of a valid self-report questionnaire frequently used to assess mood and screen for depressive symptoms in the medical population [[9-11](#_ENREF_9)]. Symptoms are rated on a scale of 0 (no symptoms) to 3 (symptoms occurring daily over the past two weeks) and summed for a total score. PHQ-9 total scores range from 0 to 27, with a score of > 10 indicating moderate or severe depression. Shown to be effective for post stroke depression screening [[12](#_ENREF_12)], a score of > 10 has 91% sensitivity and 89% specificity for major depression and 78% sensitivity and 96% specificity for any depression diagnosis. The PHQ-2 [[11](#_ENREF_11)] utilizes the first two questions of the PHQ-9 (“any depressed feelings” and “any loss of interest”). This shortened questionnaire is administered during the brief clinical screen and used to aid in the early detection of depression. A score of > 3 suggests possible depression and warrants further evaluation and treatment by the participant’s primary care team. A patient with depressive symptoms may still qualify for ICARE provided a course of care has been identified and implemented. The ICARE study staff psychologist is on call for consultation to address any concerns in this area. Study staff are instructed to contact their site-specific psychology support personnel when depressive symptoms are detected in potential candidates.

***AsTex®***

Diminished sensation is common after stroke and when present in the hand, may contribute to a reduced functional ability of the entire upper extremity. Tactile texture discrimination in the pad of the index finger is measured at each of the four evaluation time points using the AsTex® instrument [[13](#_ENREF_13)]. Ridges, decreasing in width, are aligned in parallel along the horizontal axis of the plastic rectangular instrument. Blindfolded and with the instrument placed flat on a table at midline, each participant is guided to place the pad of the less affected index finger at the most widely ridged end and to slowly slide laterally across the instrument until the ridges are no longer detected. The point where the ridges are no longer felt is recorded to the nearest 0.5 cm with the associated groove width calculated in mm. After three trials with the less affected index finger, the test is repeated for three trials with the more affected index finger.

***Confidence in Arm and Hand Movement (CAHM)***

The 20-item Confidence in Arm and Hand Movement (CAHM) scale examines self-efficacy for arm and hand function of the impaired upper extremity in individuals following stroke (Lewthwaite, Blanton, Zerigue, Winstein, Wolf—unpublished data). Items are worded to assess task-specific self-confidence for unimanual and bilateral paretic arm and hand activities typically performed in home and community contexts (e.g., “At the present time, how certain are you that you can open a large-mouth jar?”). Items are scored on a 0 (very uncertain) to100 (very certain) scale and averaged to provide a total scale score ranging from 0 to 100. Preliminary evidence of scale reliability and validity has been demonstrated in individuals with stroke and a wide range of upper extremity functional capacities. In a subsample of 72 participants enrolled in the Extremity Constraint Induced Therapy Evaluation trial [[14](#_ENREF_14)], the CAHM scale was found to be highly internally consistent, Cronbach’s alpha = .96. Test-retest reliability has been established at r =0.911 over an average 3.5 week interval in a sample of 24 individuals with stroke participating in the EXCITE trial and companion trials for stroke rehabilitation. Preliminary construct validity of the CAHM has been demonstrated in moderate correlations to measures of motor impairment in the upper extremity, laboratory-based motor performance and function, self-reported arm/hand use and quality of movement, and self-perceived activity difficulty and strength after stroke (Lewthwaite et al. unpublished data) [[15](#_ENREF_15)]. It is administered at each evaluation time point.

**Cognitive Battery**

To assess the cognitive demands of the ASAP protocol, investigators have chosen to perform a covariate analysis of cognitive abilities for all participants using a battery of five cognitive tests given at the baseline and 12-month follow-up evaluations. The battery consists of the Short Blessed Memory Test, D-KEFS Verbal Fluency Test, HVLT-R Hopkins Verbal Learning Test Revised, Color Trails Making Tests 1 & 2 and Digits Span Backwards.

***Short Blessed Test of Orientation, Concentration, and Memory***

The Short Blessed is a Test of Orientation, Concentration, and Memory consisting of 7 items. Items address orientation to month and year, short-term memory with the ability to remember a five-word phrase, and higher level processing by counting backwards from 20 to 1 and saying the months of the year in reverse order [[16](#_ENREF_16)]. The weighted sum of individual error scores is used to determine the final score. Normal cognition may be considered a score of 4 or less [[17](#_ENREF_17)].

***D-KEFS Verbal Fluency Test***

The D-KEFS Verbal Fluency Test [[18](#_ENREF_18)] assesses a participant’s ability to generate words under three different conditions: 1) letter fluency, which is the ability to name as many words as possible that begin with a specified letter of the alphabet. Letter fluency is repeated three times during the examination, each trial with a different letter of the alphabet, 2) category fluency, which is the ability to recall as many words as possible in a given category (i.e. animals or boy’s names), 3) category switching, which tests the ability of the participant to name as many items as he/she can in two given categories, while simultaneously switching between the two categories (i.e., name a piece of fruit, then a piece of furniture, then fruit, then furniture, etc.). All trials are completed in 60 seconds.

***Hopkins Verbal Learning Revised (HVLT-R))***

The Hopkins Verbal Learning Test Revised (HVLT-R) [[19](#_ENREF_19)] involves reading a list of 12 words to a participant, then asking him/her to recall as many words as possible. The exercise is repeated three times with the same list of words. Delayed recall is then tested approximately 20-25 minutes later and, without reading the list of words again, participants are asked to recall as many words from the original list that they can remember. Finally, a longer list of words is read aloud which contains words from the original list and new words. Participants are asked to say yes if the word was on the original list and no if the word was not on the original list.

***Color Trails Making Test 1 & 2***

In test one, participants are asked to connect numbered circles in a consecutive order as quickly as they can. In test two, participants are asked to connect numbered circles in consecutive order as in test one, with the added instruction of alternating colors (i.e. pink one to yellow two, to pink three to yellow four). Again, participants are asked to perform the test as quickly as they can. Errors are scored for near-misses, prompts, incorrect number sequences or color misses [[20](#_ENREF_20), [21](#_ENREF_21)]*.*

***Digit Span Backwards Test***

In the Digits Span Backward test, the examiner reads aloud a group of numbers to the participant, and then asks him/her to repeat the numbers in a backward sequence. For instance, if the examiner states ‘3-4’, a correct response from the participant would be ‘4-3’. Trials are repeated with longer sequences until the participant provides two consecutive incorrect answers.

**Activity Level Measures:**

***Motor Activity Log-MAL-28-QOM***

The the Motor Activity Log 28 (MAL-28), administered at baseline and the 12-month follow-up evaluation, is a structured self-report interview of upper extremity use [[22-24](#_ENREF_22)]. The participants are asked to use a six-point ordinal scale - 0 (unable to use arm for the activity) to 5 (use of arm is same as pre-stroke) to rank how well the more affected arm is used for 28 activities of daily living (ADL) items in the home environment. If the quality rating is determined to be between two anchors (e.g. between 2 and 3), mid-point ranking is permitted (e.g. 2.5). The tasks include functional activities such as turning a key in a lock, drinking from a cup or turning on a light switch. When administered, a demonstration video is used to provide the participant a frame of reference for the rating scale.

***EQ-5D***

The EQ5D is used as a secondary outcome measure, administered at all evaluation time points, to evaluate a participant’s perceived state of health-related quality of life and functional activity [[25](#_ENREF_25)]. The tool addresses 5 domains of functional activity (i.e. mobility; self-care; usual activity performance; pain/discomfort; and anxiety/depression). In addition, participants are asked to indicate their current perception of their health status on a visual scale rated from 0 to 100 indicating *“Worst imaginable health state”* to *“Best imaginable health state”* respectively.

**Participation Level Measures:**

***Reintegration to Normal Living Index (RNLI)***

The Reintegration to Normal Living Scale assesses the extent to which individuals with physical and cognitive conditions have resumed normal social participation [[26](#_ENREF_26)]. The 11-item RNLI surveys global function at the ICF participation level and measures participants’ satisfaction with basic self-care, home and community mobility, leisure activities, social relationships, and productive pursuits. While several response formats for the RNLI can be found in the literature, we used the 10-response Likert format in which 1 means “does not describe my situation” and 10 indicates that the statement “fully describes my situation.” [[27](#_ENREF_27), [28](#_ENREF_28)]. Item scores for the RNLI are summed and adjusted to reflect a reintegration to normal living index that ranges from 0 (no satisfaction with participation) to 100 (full satisfaction with life participation). It is administered at each evaluation time point.

***Satisfaction with Life Scale (SWLS)***

The Satisfaction with Life Scale (SWLS) is a brief evaluation tool used to determine respondents’ state of life satisfaction [[29-31](#_ENREF_29)] at each evaluation time point. The SWLS is comprised of 5 items, independent statements of Life Satisfaction, and respondents are asked to rate their agreement or disagreement with the statement on a 7-point Lickert scale, with 1 representing “Strongly Disagree” and 7 representing “Strongly Agree”

***Single item subjective quality of life (SQOL)***

The Subjective Quality of Life measure [[32](#_ENREF_32)] is a single-item rating of participant perception of overall quality of life, distinct from satisfaction with life or explicit health-related quality of life. Respondents consider *“taking everything in your life into account, please rate your current overall quality of life by placing an X on this 7-point scale.”* A visual analogue scale is anchored by the phrase “Life is very distressing” (score of 1) on the low end, “Life is great” on the high end (7) and “Life is so-so” (4) in the middle. This measure has been used in studies of individuals with disabilities resulting from a variety of diagnoses [[33](#_ENREF_33), [34](#_ENREF_34)]. It is administered at each evaluation time point.

**References**

1. Appelros P, Terent A: **Characteristics of the National Institute of Health Stroke Scale: results from a population-based stroke cohort at baseline and after one year**. *Cerebrovasc Dis* 2004, **17**(1):21-27.

2. Goldstein LB, Bertels C, Davis JN: **Interrater reliability of the NIH stroke scale**. *Arch Neurol* 1989, **46**(6):660-662.

3. Wityk RJ, Pessin MS, Kaplan RF, Caplan LR: **Serial assessment of acute stroke using the NIH Stroke Scale**. *Stroke* 1994, **25**(2):362-365.

4. Brott T, Adams HP, Jr., Olinger CP, Marler JR, Barsan WG, Biller J, Spilker J, Holleran R, Eberle R, Hertzberg V *et al*: **Measurements of acute cerebral infarction: a clinical examination scale**. *Stroke* 1989, **20**(7):864-870.

5. Andrews AW, Thomas MW, Bohannon RW: **Normative values for isometric muscle force measurements obtained with hand-held dynamometers**. *Phys Ther* 1996, **76**(3):248-259.

6. Fugl-Meyer AR, Jaasko L, Leyman I, Olsson S, Steglind S: **The post-stroke hemiplegic patient. 1. a method for evaluation of physical performance**. *Scand J Rehabil Med* 1975, **7**(1):13-31.

7. Gladstone JD DC, Black SE: **The Fugl-Meyer Assessment of Motor Recovery after Stroke: A Critial Review of its Measurement Properties**. *The American Society of Neurorehabilitation* 2002, **16**:232-240.

8. Sanford J, Moreland J, Swanson LR, Stratford PW, Gowland C: **Reliability of the Fugl-Meyer assessment for testing motor performance in patients following stroke**. *Phys Ther* 1993, **73**(7):447-454.

9. Spitzer RL, Kroenke K, Williams JB: **Validation and utility of a self-report version of PRIME-MD: the PHQ primary care study. Primary Care Evaluation of Mental Disorders. Patient Health Questionnaire**. *JAMA* 1999, **282**(18):1737-1744.

10. Kroenke K, Spitzer RL, Williams JB: **The PHQ-9: validity of a brief depression severity measure**. *J Gen Intern Med* 2001, **16**(9):606-613.

11. Kroenke K, Spitzer RL, Williams JB: **The Patient Health Questionnaire-2: validity of a two-item depression screener**. *Med Care* 2003, **41**(11):1284-1292.

12. Williams LS, Brizendine EJ, Plue L, Bakas T, Tu W, Hendrie H, Kroenke K: **Performance of the PHQ-9 as a screening tool for depression after stroke**. *Stroke* 2005, **36**(3):635-638.

13. Miller KJ, Phillips BA, Martin CL, Wheat HE, Goodwin AW, Galea MP: **The AsTex®: clinimetric properties of a new tool for evaluating hand sensation following stroke**. *Clinical Rehabilitation* 2009, **23**:1104-1115.

14. Wolf SL, Winstein CJ, Miller JP, Taub E, Uswatte G, Morris D, Giuliani C, Light KE, Nichols-Larsen D: **Effect of constraint-induced movement therapy on upper extremity function 3 to 9 months after stroke: the EXCITE randomized clinical trial**. *JAMA* 2006, **296**(17):2095-2104.

15. Stewart JC: **Planning of unconstrained reach actions after unilateral sensorimotor stroke.** *Unpublished doctoral dissertation.* Dissertation Abstracts International: University of Southern California; 2010.

16. Katzman R, Brown T, Fuld P, Peck A, Schechter R, Schimmel H: **Validation of a short orientation-memory concentration test of cognitive impairment**. *A J Psychiatry* 1983, **140**:734-739.

17. Morris JC, Heyman A, Mohs RC, Hughes JP, van Belle G, Fillenbaum G, Mellits ED, Clark C: **The Consortium to Establish a Registry for Alzheimer's Disease (CERAD). Part I. Clinical and neuropsychological assessment of Alzheimer's disease**. *Neurology* 1989, **39**(9):1159-1165.

18. Delis DC, Kaplan E, Kramer JH: **The Delis-Kaplan executive function system**. San Antonio: The Psychological Corporation; 2001.

19. **Hopkins Verbal Learning Test-Revised**. In*.*: PAR Psychological Assessment Resources, Inc.; 2001.

20. Army US: **Manual of direction and scoring**. In*.* Edited by Department W. Adjutant General's office; 1944.

21. Reitan R, Wolfson D: **The Halstead-Reitan neuropsychological test battery: theory and clinical interpretation 2nd edition**. Tuscon, AZ: Neuropsychology Press; 1993.

22. Uswatte G, Taub E, Morris D, Light K, Thompson PA: **The Motor Activity Log-28: assessing daily use of the hemiparetic arm after stroke**. *Neurology* 2006, **67**(7):1189-1194.

23. van der Lee JH, Beckerman H, Knol DL, de Vet HC, Bouter LM: **Clinimetric properties of the motor activity log for the assessment of arm use in hemiparetic patients**. *Stroke* 2004, **35**(6):1410-1414.

24. Taub E, Miller NE, Novack TA, Cook EW, 3rd, Fleming WC, Nepomuceno CS, Connell JS, Crago JE: **Technique to improve chronic motor deficit after stroke**. *Arch Phys Med Rehabil* 1993, **74**(4):347-354.

25. **EuroQuol--a new facility for the measurement of health-related quality of life. The EuroQol Group**. *Health Policy* 1990, **16**(3):199-208.

26. Wood-Dauphinee SL, Opzoomer MA, Williams JI, Marchand B, Spitzer WO: **Assessment of global function: The Reintegration to Normal Living Index**. *Arch Phys Med Rehabil* 1988, **69**(8):583-590.

27. Stark SL, Edwards DF, Hollingsworth H, Gray DB: **Validation of the Reintegration to Normal Living Index in a population of community-dwelling people with mobility limitations**. *Arch Phys Med Rehabil* 2005, **86**(2):344-345.

28. Daneski K, Coshall C, Tilling K, Wolfe CDA: **Reliability and validity of a postal version of the Reintegration to Normal Living Index, modified for use with stroke patients**. *Clinical Rehabilitation* 2003, **17**(8):835-839.

29. Diener E, Emmons RA, Larsen RJ, Griffin S: **The Satisfaction With Life Scale**. *J Pers Assess* 1985, **49**(1):71-75.

30. Pavot W, Diener E: **Review of the Satifaction With Life Scale**. *Psychol Assess* 1993, **5**(2):164-172.

31. Arrindell WA, Meeuwesen L, Huyse FJ: **The Satisfaction With Life Scale (SWLS): Psychometric properties in a non-psychiatric medical outpatients sample**. *Pers Individ Dif* 1991, **12**(2):117-123.

32. Kemp BJ, Ettelson D: **Quality of life while living and aging with a spinal cord injury and other impairments**. *Top Spinal Cord Inj Rehabil* 2001, **6**(3):116-127.

33. Kemp BJ: **What the rehabilitation professional and the consumer need to know**. *Phys Med Rehabil Clin N Am* 2005, **16**(1):1-18, vii.

34. Kemp BJ, Mosqueda L: **Aging with a disability: What the clinician needs to know**. Baltimore: Johns Hopkins University Press; 2004.
